# Extreme Ultraviolet Multilayer Defect Profile Parameters Reconstruction via Transfer Learning with Fine-Tuned VGG-16

**DOI:** 10.3390/mi16050541

**Published:** 2025-04-30

**Authors:** Hala Mohammad, Jiawei Li, Bochao Li, Jamilu Tijjani Baraya, Sana Kone, Zhenlong Zhao, Xiaowei Song, Jingquan Lin

**Affiliations:** 1 School of Physics, Changchun University of Science and Technology, Changchun 130022, China; 2019300091@mails.cust.edu.cn (H.M.);; 2 Zhongshan Research Institute, Changchun University of Science and Technology, Zhongshan 528400, China; 3 Chongqing Research Institute, Changchun University of Science and Technology, Chongqing 401120, China

**Keywords:** EUV lithography, multilayer defects, transfer learning, fine-tuning, VGG-16

## Abstract

Extracting defect profile parameters from measured defect images poses a significant challenge in extreme ultraviolet (EUV) multilayer defect metrologies, because these parameters are crucial for assessing defect printing behavior and determining appropriate repair strategies. This paper proposes to reconstruct defect profile parameters from reflected field intensity images of a phase defect assisted by transfer learning with fine-tuning. These images are generated through simulations using the rigorous finite-difference time-domain (FDTD) method. The VGG-16 pre-trained model, known for its robust feature extraction capability, is adopted and fine-tuned to map the intensity images to the defect profile parameters. The results demonstrate that the proposed approach accurately reconstructs multilayer defect profile parameters, thus providing important information for mask repair strategies.

## 1. Introduction

EUV lithography is a key technology for the manufacturing of next-generation integrated circuits [1]. An EUV mask blank featuring a reflective Mo/Si multilayer film plays a crucial role in this process [2,3]. Defects arising from particle deposition within or beneath the multilayer or from pits on a blank substrate [4,5] can disrupt the layer structure, causing changes in the amplitude and phase of the reflected field [6]. These changes can negatively impact the lithographic process [6,7,8]. Since achieving completely defect-free multilayer masks is extremely challenging in practice, strategies for defect mitigation are currently in use [9]. These strategies involve modifying the mask absorber pattern with repair tools to compensate for adjacent multilayer defects and using the absorber pattern strategically to cover defects [10,11,12]. The successful application of these strategies relies on precise defect detection and characterization [13].

To detect and characterize possible defects, specialized inspection machines and metrology tools are used [14]. These devices capture images of defects and extract key characteristics, such as profile information. For instance, the micro-coherent scatterometry microscope (micro-CSM) system and the atomic force microscope (AFM) are capable of measuring the surface profiles of defects [15,16,17]. Nevertheless, both AFM and micro-CSM lack the capability to non-destructively characterize the internal profiles of multilayer defects, making them insufficient for accurate defect repair [17,18,19,20]. To overcome this limitation, various nondestructive approaches were proposed to indirectly characterize the three-dimensional profiles of multilayer defects. Methods based on the Stearns growth model [6,21] and the level-set multilayer growth model [22] were developed to reconstruct the three-dimensional profiles of multilayer defects. These approaches involve combining AFM measurements of the top profiles of phase defects with deposition-based growth models to estimate the bottom profiles. However, these models are highly dependent on deposition conditions, which affects their applicability [23].

Recently, mapping functions from defect deformation information (e.g., aerial images) to defect geometric parameters have been constructed to characterize multilayer defects [3]. Xu et al. [24] applied the transport-of-intensity equation (TIE) [25,26] to retrieve phase information from simulated projection images at various focus positions and used principal component analysis (PCA) to simplify the intensity and phase data representation. An artificial neural network (ANN) was used to analyze the correlation between the PCA coefficients and defect geometric parameters. Similarly, Dou et al. [27] utilized partial least square regression (PLSR) [28] to map the phase deformation properties retrieved from scattering images to defect geometric parameters. To improve characterization accuracy, Chen et al. [3] implemented an inception-based neural network with cycle-consistent learning. Cheng et al. [17] introduced an approach that leverages aerial images’ complex amplitudes. Fourier ptychography (FP) was employed to retrieve the phase information from a defective mask blank, and a convolutional neural network based on dilated residual networks (DRNs) was used to correlate the retrieved amplitudes and phases with the defect profile parameters. Building on these efforts, Zheng et al. [20] developed an artificial neural network (ANN) framework that incorporated aerial images collected at various illumination angles. By integrating generative adversarial networks (GANs), their method achieved highly accurate defect characterization. Another advanced approach by Li et al. [29] integrated EUV photoemission electron microscopy (EUV-PEEM) images with transfer learning using ResNet18, enabling the accurate reconstruction of the phase defect three-dimensional morphology.

Aerial imaging-based multilayer defect characterization, while effective, presents several challenges. The data acquisition process is complex, not always accessible in all research facilities, and often requires specialized settings, such as multiple illumination angles. Moreover, it is time-consuming and demands a large dataset for accurate defect characterization. For instance, the GAN-based approach required 5120 training samples for each defect type and over 60 min of training to achieve a high accuracy [20]. Although the EUV-PEEM-based approach reduces the dataset size and training time, it still necessitates post-processing and further calculations to generate EUV-PEEM images following the simulation of the reflected field [29]. These limitations highlight the need for a more efficient approach to characterize multilayer defects.

In this paper, we propose a novel approach to reconstruct multilayer defect profile parameters assisted by transfer learning with a fine-tuned VGG-16 model [30]. The defect profile parameters considered in this study include the top height (h_top_), top width (W_top_), and bottom size (S_bot_) of the multilayer defect. By using a pre-trained VGG-16 model, our approach significantly reduces the computational costs and eliminates lengthy training processes. This method enables the accurate reconstruction of defect profile parameters with a smaller dataset, offering a more efficient solution for EUV mask blank defect characterization. The results demonstrate that this approach can efficiently reconstruct defect profile parameters, making it a promising alternative to the existing data-intensive approaches.

## 2. Theoretical Model

This section introduces the simulation process for the reflected field intensity from defective blank masks using the finite-difference time-domain (FDTD) method. Additionally, it describes the application of transfer learning with a pre-trained VGG-16 network, where its final layers are fine-tuned for the specific task of multilayer defect profile parameters reconstruction.

### 2.1. Reflected Field Intensity Simulation from a Defective Blank Mask

**Figure 1:**
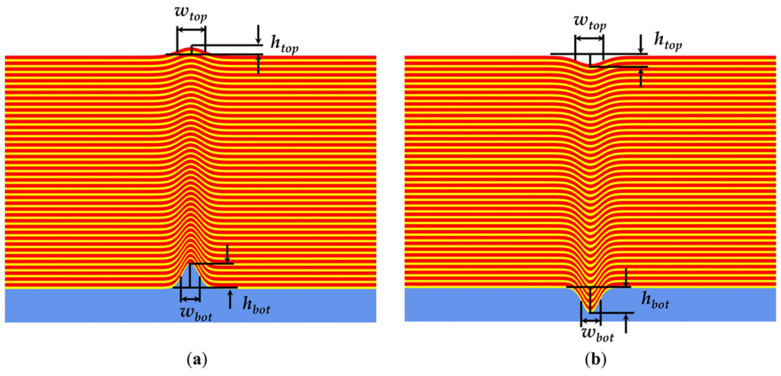
EUV blank mask with (**a**) bump and (**b**) pit defects.

In this study, we adopt a Gaussian-shaped defect model to characterize the profile of multilayer defects, as it effectively represents the natural defect profile [17]. The defects are assumed to be rotationally symmetric [24], with the height at the bottom of the multilayer (h_bot_) and the width at the bottom of the multilayer (W_bot_) being equal (i.e., h_bot_ = W_bot_ = S_bot_). Here, S_bot_ refers to the bottom size. This assumption simplifies the investigation and reduces the computational time required for a fully rigorous calculation of the reflected field. Both bump and pit defects are considered. Figure 1 shows the profiles of the two defects on a blank mask substrate. Although these defects, which cause multilayer deformation, initially have distinct profiles, they are gradually smoothed into a relatively regular profile when covered by deposited Mo/Si multilayers [20,31].

The intensity images are simulated using the rigorous FDTD method, which is an important approach for numerically calculating electromagnetic fields [32]. The simulation settings are as follows: The size of the simulation region is set to 300 nm × 300 nm. A smaller mesh size is used, with ∆x = 1.5 nm, ∆y = 0.25 nm, and ∆z = 1.5 nm. A TE-polarized plane wave of 13.5 nm illuminates the mask blank at an incident angle of 6° along the negative y-axis. The blank mask consists of 40 bilayers of 2.78 and 4.17 nm thick Mo and Si, respectively. Table 1 summarizes the simulation settings used.

Considering the defect profile parameters, h_top_ is sampled from 0.5 to 5 nm at 0.5 nm intervals, W_top_ from 40 to 70 nm, and S_bot_ from 10 to 40 nm, both at 5 nm intervals. The sampled values of h_top_, W_top_, and S_bot_ yield 490 combinations of bump defects and 490 combinations of pit defects. To establish the dataset, a fully rigorous simulation is conducted for each combination.

The profile parameters for the intensity images simulated using the rigorous FDTD method were primarily selected based on prior work by Xu et al. on multilayer defect profile parameters reconstruction [24] and further refined for computational efficiency by adjusting the sampling ranges for the profile parameters. Specifically, the upper limits for the top width and bottom size of the defects were reduced.

### 2.2. Transfer Learning with Fine-Tuning

In lithography, when deep learning methods are applied, a common challenge is the requirement for large training datasets, which are often unavailable [33]. Therefore, there is a need to develop high-performance models that can be trained using limited available data. This paves the way for another deep learning strategy, transfer learning, a promising technique for addressing data scarcity issues. Transfer learning utilizes a pre-trained model (source model) trained on a large dataset (source dataset) to enhance learning for a target task with limited training data [34,35,36]. For instance, in our case, with a small dataset of only 490 intensity images, we can leverage transfer learning and use a pre-trained model such as VGG-16, which was originally trained on a large dataset (e.g., ImageNet), to adapt the generalizable features learned from the large dataset for our task of EUV multilayer defect profile parameters reconstruction with limited data available.

**Figure 2:**
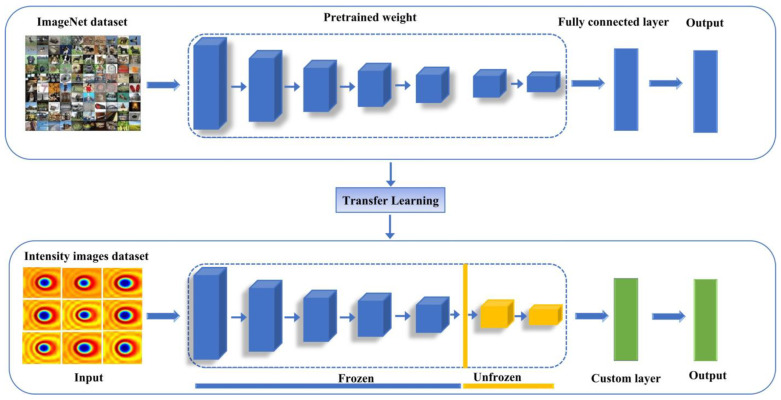
Transfer learning with a fine-tuning approach.

While large image datasets are typically from general domains, the target dataset may differ in visual representation, making the direct application of learned features less effective. To adapt a pre-trained model to a new task, certain layers are retrained, while others remain unchanged (frozen) [37]. This adaptation process is typically achieved through a fine-tuning approach [37,38]. During fine-tuning, the final layers of a deep neural network are typically adjusted (unfrozen), whereas the initial layers retain their pre-trained weights. This method reduces the number of trainable parameters, thereby mitigating the risk of overfitting. The motivation for this approach stems from dataset limitations and empirical evidence: lower network layers capture generic features applicable to multiple tasks, while higher layers learn more task-specific representations [37,39,40]. Figure 2 illustrates the transfer learning process with fine-tuning. As fine-tuning tailors the model to the target task, it enhances performance and is widely employed in CNN-based transfer learning for data-limited domains [41].

### 2.3. Defect Profile Parameters Reconstruction Model

To obtain the defect profile parameters from the intensity distribution images, it is necessary to establish a mapping between them. In this study, we employ the transfer learning technique using the pre-trained VGG-16 model, leveraging its robust feature extraction and learning capabilities [30] and fine-tuning it to create this mapping. Among the various pre-trained models, the VGG-16 model was selected for its ease of implementation and relatively small number of parameters, which results in a faster learning network [42].

**Figure 3:**
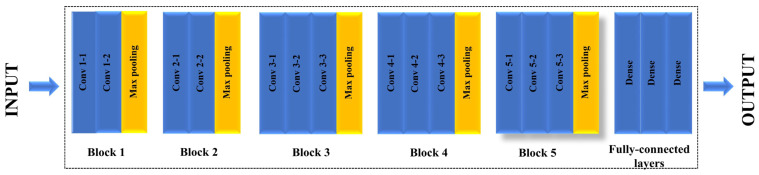
VGG-16 pre-trained model architecture.

VGG-16 is a CNN model developed by the Visual Geometry Group (VGG) of the University of Oxford [43] and the winner of the 2014 ILSVRC object identification algorithm [43,44]. It is a 16-layer deep neural network structured into five blocks followed by a set of fully connected layers [45]. The standard model architecture is shown in Figure 3. The first two blocks (Blocks 1 and 2) contain two convolutional layers each, whereas the remaining blocks (Blocks 3, 4, and 5) contain three convolutional layers each [45,46]. All convolutional layers use a 3 × 3 kernel size with a ReLU activation function applied after each operation [47]. The use of a smaller kernel size reduces the total number of parameters and helps mitigate the risk of overfitting—an important consideration when training on smaller datasets [42]. At the end of each block, a 2 × 2 max pooling layer is used for downsampling. The final segment of the VGG-16 network consists of three fully connected layers, with the final output typically obtained using the softmax function [30,47].

**Figure 4:**
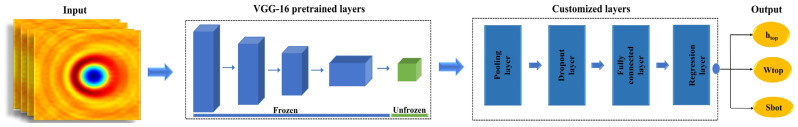
Pre-trained VGG-16 model with customized layers added on top for multilayer defect profile parameters reconstruction.

For multilayer defect profile parameters reconstruction, we use the pre-trained VGG-16 model without its fully connected layers. A customized layer set is then added for regression. This includes a global average pooling layer to downsample the feature maps [48], a dropout layer to reduce overfitting [49], and a fully connected (dense) layer with 512 neurons. Glorot Uniform is used for weight initialization [50], and L2 regularization is applied to penalize large weights and prevent overfitting [51]. This dense layer is followed by a final output layer with a single neuron and a linear activation function to output a single prediction. Figure 4 shows the pre-trained model with the added customized layers. To fine-tune the model to the new task, we unfreeze the last block in the base model while keeping the earlier blocks frozen to retain the pre-trained weights. To improve the reconstruction accuracy, three separate neural networks are created using the same base architecture, but with slightly different hyperparameter settings, each specializing in predicting one specific defect profile parameter (h_top_, W_top_, and S_bot_).

## 3. Results and Discussion

### 3.1. Analysis of the Reflected Field Intensity Images

**Figure 5:**
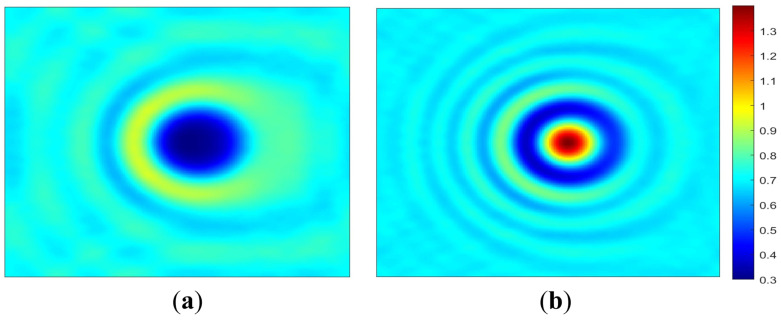
Reflected field intensity distribution for an EUV mask blank with (**a**) bump and (**b**) pit defects. Both bump and pit defects have h_top_ = 0.5 nm, W_top_ = 40 nm, and S_bot_ = 20 nm.

This section shows how the reflected field intensity changes with respect to the three defect profile parameters (h_top_, W_top_, and S_bot_). It is confirmed in this section that the impact of the multilayer defect is closely related to the defect profile parameters. Changes in these parameters result in varying effects on the reflected field intensity. Figure 5 shows the reflected field intensity distribution images for an EUV mask blank with bump (Figure 5a) and pit (Figure 5b) defects.

**Figure 6:**
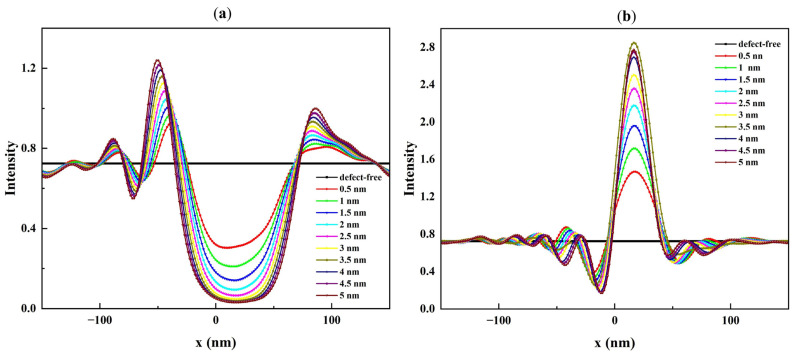
Cross-section cuts of the intensity distribution along the x-axis of (**a**) bump and (**b**) pit defects with different top heights ranging from 0.5 to 5 nm, top widths = 40 nm, and bottom sizes = 20 nm.

**Figure 7:**
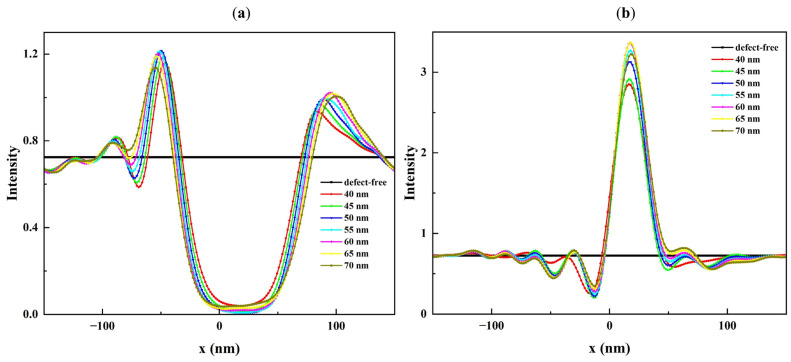
Cross-section cuts of the intensity distribution along the x-axis of (**a**) bump and (**b**) pit defects with different top widths ranging from 40 to 70 nm, top heights = 3.5 nm, and bottom sizes = 20 nm.

**Figure 8:**
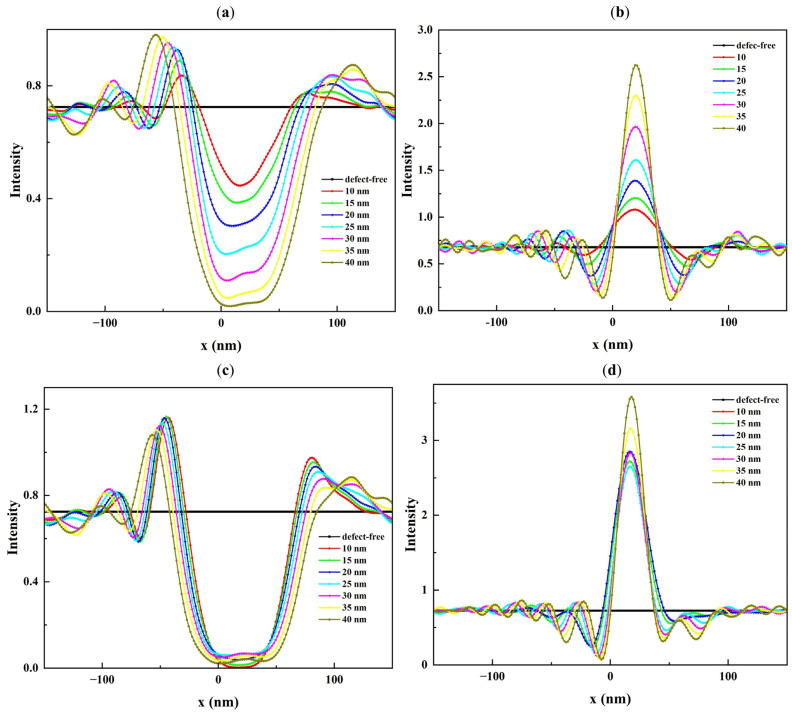
Cross-section cuts of the intensity distribution along the x-axis of (**a**) bump and (**b**) pit defects with top heights of 0.5 nm, and (**c**,**d**) bump and pit defects with top heights of 3.5 nm. All defects have different bottom sizes ranging from 10 to 40 nm and top widths of 40 nm.

Due to the 6° illumination angle of the mask blank, the center of the intensity images shifts accordingly, with the most pronounced impact occurring in the central region. As seen in Figure 5, the central region of these images is significantly influenced by the presence of defects. The bump defect, shown in Figure 5a generates a local intensity minimum, whereas the pit defect, shown in Figure 5b, causes a local maximum. The magnitude of the local intensity variation changes with the defect profile parameters. Figure 6, Figure 7 and Figure 8 show cross-section cuts along the *x*-axis of the intensity distribution for bump and pit defects with varying top heights (h_top_), top widths (W_top_), and bottom sizes (S_bot_).

It is clear from Figure 6 that the intensity minima for a rotationally symmetric bump defect and the intensity maxima for a rotationally symmetric pit defect are sensitive to h_top_, especially when the top height is below 3.5 nm. While the range of h_top_ is small, even slight changes (0.5 nm) in h_top_ result in significant changes in the reflected field intensity. While W_top_ and S_bot_ have a larger value range compared to h_top_, their influence on the reflected field intensity is lower. The results show that W_top_ has only a small impact on the observed intensity minima and maxima (Figure 7a,b). Furthermore, S_bot_ has a certain impact on the reflected field intensity when h_top_ is less than or equal to 3.5 nm. Figure 8a,b show examples of this effect for S_bot_ when h_top_ = 0.5 nm. As h_top_ increases, the defect causes a stronger deformation of the multilayer. In the case of a bump defect, the intensity drops to a small value, while in the case of a pit defect, the intensity increases to a large value and becomes less sensitive to variations in S_bot_ (Figure 8c,d).

### 3.2. Model Performance Evaluation

To reconstruct the multilayer defect profile parameters, a VGG-16 model, as shown in Figure 4, is designed to map the intensity images to the defect profile parameters. The intensity images serve as the input for the VGG-16 model. Model building, training, and testing are performed using TensorFlow as the backend with Keras as the high-level API within the Google Colab environment with an NVIDIA A100-SXM4-40GB GPU. The Adam optimizer is used as the gradient descent for training. During the model training process, the mean absolute error (MAE) is employed as the loss function. The average relative error (ARE) and MAE are adopted as the evaluation metrics to assess the accuracy of the profile parameters reconstruction. ARE and MAE are defined as follows [17]:

(1) $$ARE=\;\left(100\times \frac{1}{n}\sum_{i=1}^{n}\;\left|\frac{P_{rec}-P_{def}}{P_{def}}\right|\right)\%,\;$$

(2) $$MAE=\left(\frac{1}{n}\sum_{i=1}^{n}\left|P_{rec}-P_{def}\right|\right),\;$$

where *n* denotes the total number of samples in the testing set, *P_rec_* represents the reconstructed parameter, and *P_def_* is the defined parameter.

Given that the train–test split ratio can influence the prediction performance of the model, it is essential to choose a well-balanced split that ensures a sufficiently large training set and a representative testing set. This balance enables the model to capture complex patterns effectively while accurately evaluating its performance [52]. Based on our experiments, we found that using 370 images for training and 120 images for testing provided the best performance. This ratio strikes an optimal balance between the training and testing sets, leading to the most reliable model performance. Both the training and testing sets consist of intensity images of the blank mask with defects, along with the corresponding defect profile parameters: h_top_, W_top_, and S_bot_. Table 2 presents the hyperparameter settings used to train these models.

**Figure 9:**
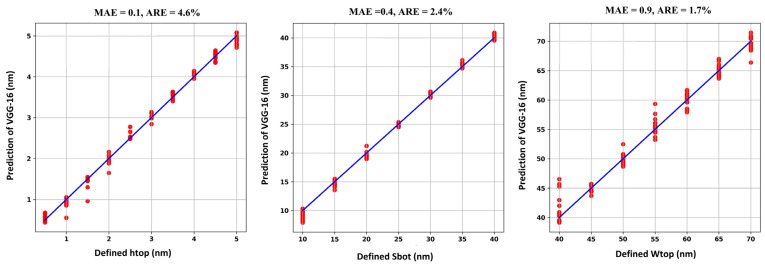
EUV multilayer defect profile parameters reconstruction results for the bump defect.

**Figure 10:**
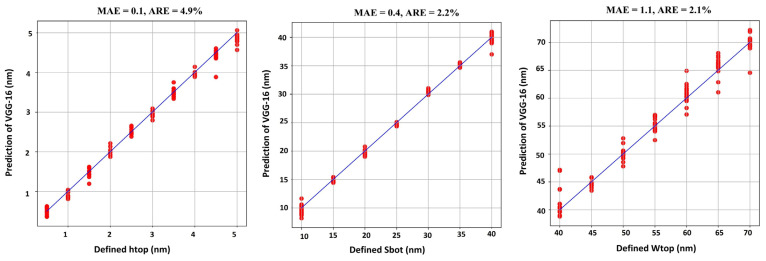
EUV multilayer defect profile parameters reconstruction results for the pit defect.

The reconstruction results of the defect profile parameters of the bump and pit defects are shown in Figure 9 and Figure 10, respectively. The x-axis represents the defined values for each parameter, while the y-axis represents the predicted values generated by the VGG-16 model. Ideally, if the model predictions are perfect, the red dots will align exactly along the straight blue line.

From the established dataset, 370 images are used for training, and the remaining 120 images are used to test the model’s performance. Each model takes about 4 min to train. As observed in Figure 9 and Figure 10, the trained VGG-16 model effectively reconstructs the profile parameters of the defects, with both defect types exhibiting an MAE of less than 1 nm and an average error rate of 2.9% and 3.06%, respectively. Table 3 presents the reconstruction accuracy for the bump and pit defects in terms of MAE and ARE.

Compared to previous work using CNN with cycle-consistent learning and the inception module [3], our method reduces the error rate from 3.02% to 2.9% for the bump defect. While the improvement is modest, our approach achieves better accuracy despite the already low error rate of the CNN + inception model (3.02%). Other methods, such as Fourier ptychographic imaging (FPI) + DRN [17] and DRN+GAN [20], have reported superior reconstruction accuracy, but they required significantly larger datasets and longer training times. For instance, the inception-based CNN required 3200 aerial images [3], while FPI + DRN [17] and DRN + GAN [20] required a total of 5120 bump and 5120 pit defect aerial images, which were collected at multiple illumination angles. In comparison to the most recent work using transfer learning with ResNet-18 and EUV-PEEM images [29], our method demonstrated almost the same training time and used a comparable dataset size. While their work achieved superior accuracy for bump and pit defects, reporting error rates of 1.37% and 1.39%, respectively, it required additional post-processing and further calculations to generate EUV-PEEM images following the simulation of the reflected field. This added complexity introduces additional steps into the process. Table 4 shows a comparison between previous work on EUV multilayer defect profile parameters reconstruction using deep learning approaches.

One of the key advantages of our approach is its efficiency in terms of both data requirements and training time. Our model achieves high accuracy using only 490 samples per defect type, which is a substantial reduction in dataset size, and these samples were collected at a single illumination angle (6°). This reduces the burden of dataset collection, making our method more suitable for scenarios where acquiring large amounts of training data is challenging. Additionally, it significantly reduces the training time. While previous methods required several thousand seconds for training, our model completes the process in approximately 720 s, effectively reducing the computation time by an order of magnitude. Moreover, our proposed model offers the capability to non-destructively characterize the internal profile of the defect, thus surpassing conventional approaches for multilayer defect characterization.

The current model provides a foundational proof-of-concept for defect profile parameters reconstruction and validates the feasibility of the proposed approach for isolated defects (single pits or bumps) under the assumption of linear optical behavior. However, real-world EUV photomasks often present more complex defect scenarios, such as coexisting pits and bumps or multiple defects of the same type. Additionally, while our current study primarily focuses on defects occurring at the substrate level of EUV mask blanks, which are the most prevalent, accounting for an average of 75% of the defects observed at the mask blank level [53], defects can also arise within the multilayer. This aspect is equally critical. Furthermore, higher energies can lead to more pronounced multiphoton absorption effects, which could alter the optical response of the material [54]. These nonlinear effects could impact the reflection patterns used for defect profile parameters reconstruction, potentially affecting the accuracy of the model’s predictions. Moreover, the focus position variation can also influence the intensity distribution and defect characterization, as local image intensity is nonlinear with respect to focus [24]. This could further affect the accuracy of defect profile parameters reconstruction. Addressing these challenges will enable the model to handle more intricate defect scenarios and improve its accuracy in practical applications.

## 4. Conclusions

This study presents a novel approach for multilayer defect profile parameters reconstruction using transfer learning with a fine-tuned VGG-16 model. By leveraging the robust feature extraction capabilities of the pre-trained VGG-16 model and fine-tuning it to map the reflected field intensity images to the defect profile parameters, the approach demonstrates its ability to accurately reconstruct multilayer defect profile parameters from simulated intensity images. The proposed method provides a balanced trade-off by maintaining an accurate profile parameters reconstruction while significantly reducing the data requirements and training time. We believe that this approach paves the way for rapid and precise EUV mask defect compensation in semiconductor manufacturing.

Future work will focus on refining the model to address more complex defect scenarios, including coexisting pits and bumps or multiple defects of the same type. In addition, defects that can arise within the multilayer will also be considered, as they are also critical to the lithographic process. Moreover, we will examine nonlinear behaviors, such as multiphoton absorption effects, and the impact of focus position variation. Incorporating focus position variation could enhance the model’s accuracy and robustness, particularly in real-world settings where defects are often observed at multiple focus levels.

## Figures and Tables

**Table 1:** Parameters setting in the simulation using FDTD.

Object	Parameter	Value
Simulation region	Size	300 × 300 nm
	Mesh size	∆x = 1.5 nm, ∆y = 0.25 nm, ∆z = 1.5 nm
Illumination	Angle	6°
	Polarization	TE-polarized
	Direction	Negative y-axis
	Wavelength	13.5 nm
Mask blank	Number of bilayers	40 bilayers of Mo and Si
	Mo-Si thickness	4.17 nm thick Si, 2.78 nm thick Mo
	substrate thickness	50 nm thick SiO_2_
	Mo-Si properties	For Si: n = 0.999, K = 0.00182 For Mo: n = 0.923, K = 0.00622

**Table 2:** Models training hyperparameters setting. The mark (″) indicates that the value in that row is the same as the corresponding value in the row above it.

Defect Type		Batch Size	Epochs	Dropout Rate	Learning Rate	Regularization Factor
Bump	h_top_	10	150	0.2	0.000020	0.025
	w_top_	″	″	″	0.000025	0.030
	S_bot_	″	″	″	0.000025	0.010
Pit	h_top_	″	″	″	0.000020	0.025
	w_top_	″	″	″	0.000025	0.025
	S_bot_	″	″	″	0.000020	0.025

**Table 3:** Performance evaluation results for multilayer defect profile parameters reconstruction.

Defect Type	h_top_		W_top_		S_bot_	
	MAE (nm)	ARE (%)	MAE (nm)	ARE (%)	MAE (nm)	ARE (%)
Bump	0.1	4.6	0.9	1.7	0.4	2.4
Pit	0.1	4.9	1.1	2.1	0.4	2.2

**Table 4:** Comparison between different deep learning-based approaches for multilayer defect profile parameters reconstruction.

Approach	Data Type	Dataset Size per Defect Type	Training Time (s)	Accuracy (ARE %)	Data Collection Requirements
CNN + cycle-consistent learning + inception module [3]	Aerial images	2000 for bump	2160	3.02%	No
Fourier ptychographic imaging (FPI) + DRN [17]	Aerial images	5120 for bump 5120 for pit	//	~ 2.1% for bump ~ 1.9% for pit	Yes
DRN + GANs [20]	Aerial images	5120 for bump 5120 for pit	3976	1.37% for bump 1.39% for pit	Yes
ResNet-18 [29]	EUV-PEEM	360 for bump 360 for pit	∼900	1.37% for bump 1.39% for pit	Yes
VGG-16 (this work)	Intensity images	490 for bump 490 for pit	720	2.9% for bump 3.06% for pit	No

## Data Availability

The raw data supporting the conclusions of this article will be made available by the authors upon request.
